# Inhibitory Control Across the Preschool Years: Developmental Changes and Associations with Parenting

**DOI:** 10.1111/cdev.13426

**Published:** 2020-08-07

**Authors:** Sanne B. Geeraerts, Joyce J. Endendijk, Maja Deković, Jorg Huijding, Kirby Deater‐Deckard, Judi Mesman

**Affiliations:** ^1^ Utrecht University; ^2^ Umass Amherst; ^3^ Leiden University

## Abstract

The normative developmental course of inhibitory control between 2.5 and 6.5 years, and associations with maternal and paternal sensitivity and intrusiveness were tested. The sample consisted of 383 children (52.5% boys). During four annual waves, mothers and fathers reported on their children’s inhibitory control using the Children's Behavior Questionnaire. During the first wave, mothers’ and fathers’ sensitivity and intrusiveness were observed and coded with the Emotional Availability Scales. Inhibitory control exhibited partial scalar invariance over time, and increased in a decelerating rate. For both mothers and fathers, higher levels of sensitivity were associated with a higher initial level of children's inhibitory control, whereas higher levels of intrusiveness predicted a slower increase in children's inhibitory control.

Developing the capacity for self‐regulation, that is, the ability to automatically or deliberately modulate affect, behavior, and cognition (Karoly, 1993), is an important task in childhood and adolescence. Higher levels of self‐regulation are related to fewer mental health problems, and better academic performance (Bull, Espy, & Wiebe, 2008; Olson, Sameroff, Kerr, Lopez, & Wellman, 2005). One key component of self‐regulation is inhibitory control, that is, the ability to plan and suppress responses (Rothbart, Ahadi, Hershey, & Fisher, 2001). Understanding the normative developmental course of inhibitory control, and the factors that may predict this development, can help professionals in detecting developmental problems early on and identifying intervention targets with parents. Therefore, the aim of the current study is to model the development of inhibitory control in Dutch preschool children between the ages of 2.5 and 6.5 years, and to examine whether parental sensitivity and intrusiveness predict the course of development.

## Development of Inhibitory Control

Early displays of inhibitory control are already seen during the first year of life. Even 8‐month‐old children can prevent or stop behaviors in response to their parent’s requests (Kochanska, Tjebkes, & Fortnan, 1998). Over the preschool years, inhibitory control develops rapidly. Prior studies have tracked this development by looking at mean‐level changes across a variety of lab tasks (Dennis, Brotman, Huang, & Gouley, 2007; Klenberg, Korkman, & Lahti‐Nuuttila, 2001; Schoemaker, Bunte, Espy, Deković, & Matthys, 2014). These studies indicate that inhibitory control develops especially fast during the early preschool years. Specifically, a study with a group of predominantly clinically referred preschool children with externalizing problems (age between 3.5 and 5.6 years at the first wave) demonstrated that inhibitory control improved over a course of 18 months, and that this development was the strongest between the age of 3.5 and 4.5 years (Schoemaker et al., 2014). Similarly, a study with 75 children at risk for conduct problems indicated that inhibitory control increased rapidly between 4 and 5 years, and that this increase levels off between 5 and 6 years (Dennis et al., 2007). Another study found similar results for simple inhibition (suppressing a dominant response) in a sample with children who were not at risk in their development (Lengua et al., 2015). Lastly, a study with a normative sample between 3 and 12 years of age indicated that the ability for simple inhibition improved until the age of 6 years, and the ability for complex inhibition (suppressing a dominant response and activating a subdominant response) improved until the age of 7 years (Klenberg et al., 2001).

Despite a handful of studies on the early development of inhibitory control as assessed with lab tasks, we know little about the development of inhibitory control as expressed in daily life. Although studies conducted with information obtained in lab settings are highly valuable, data provided by parents on acts of inhibitory control in real‐world situations (e.g., are children capable of waiting in line, do they obey instructions) add to our knowledge by providing a more ecologically valid measure. The most widely used questionnaire for parents to report on inhibitory control is the Children’s Behavior Questionnaire (CBQ; Rothbart et al., 2001). A meta‐analysis on the usefulness of various inhibitory control measures across age, based on cross‐sectional data, concluded that the CBQ inhibitory control subscale is useful to measure individual differences in inhibitory control within a 6‐year age range (i.e., from age 2 to 8 years), whereas lab tasks were on average only useful for detecting individual differences within a 2.49‐year age range (Petersen, Hoyniak, McQuillan, Bates, & Staples, 2016).

Although useful across a wide age range, in the meta‐analysis on inhibitory control measures it appears that there is only a modest increase in scores on the CBQ inhibitory control scale across age (Petersen et al., 2016). Longitudinal research on the CBQ inhibitory control scale indicates a decelerating increase between 2 and 7.5 years (Chang, Shaw, Dishion, Gardner, & Wilson, 2014; Moilanen, Shaw, Dishion, Gardner, & Wilson, 2010). Overall, studies thus far indicate that findings on the development of inhibitory control measured with lab tasks are not necessarily generalizable to parent‐reported inhibitory control, as parent‐reported inhibitory control may increase less during the preschool years.

As the CBQ was developed to measure a temperament related construct, it may be argued that it is not designed to detect mean‐level change. However, whereas earlier theories regarding temperament underscored the longitudinal stability of temperament, Rothbart and colleagues have argued against this conceptualization (see also Putnam & Stifter, 2008 for a review on this matter). Rothbart reasoned that developmental changes in temperament are likely, due to the emergence of new skills, other expressions of behavior, and because temperament is an open system that is influenced by interactions with the environment (Rothbart, 2012). Within this framework, inhibitory control and other aspects that relate to effortful control develop substantially in the preschool years, both through the emergence of new skills and the improvement of existing skills (Rothbart & Rueda, 2005). It is therefore to be expected that inhibitory control measured through the CBQ also increases in mean‐level over time, but not necessarily in the same manner as inhibitory control as assessed through lab tasks. Development may for instance be slower, because children do not necessarily immediately implement the cognitive skills that are measured with lab tasks in their daily life. Exclusively relying on lab tasks for measuring growth in inhibitory control may create unrealistic expectations regarding the development that children demonstrate in inhibitory control over the preschool years. It is therefore important to examine parent reports of inhibitory control as well. Moreover, most studies on the development of inhibitory control (both parent‐rated and lab‐based tasks) have utilized at‐risk samples. Less is known about how these results generalize to children who are not at risk in their development.

Another limitation of previous work on parent‐reported inhibitory control concerns the absence of longitudinal measurement invariance testing. Younger children may not only show lower levels of inhibitory control (i.e., mean‐level change) but may also show different behaviors that indicate their level of inhibitory control (i.e., conceptual change). Potential conceptual changes may be due to preschoolers significant development in multiple domains, such as language and motor development, that interacts with the way in which inhibitory control is manifested (e.g., Hughes & Graham, 2002), as well as developmental changes in contexts. An important contextual milestone to consider is the transition to school, which happens at the age of 4 years in the Netherlands. This transition brings a variety of changes in children’s environments, including different expectations when it comes to following instructions, remaining seated, and inhibiting unwanted behaviors. As a result, observed changes in inhibitory control can be confounded by other developmental processes.

Conceptual changes in inhibitory control can hinder interpretations regarding mean‐level developmental changes in inhibitory control. To test for possible conceptual changes in inhibitory control, longitudinal measurement invariance should be examined. Measurement invariance ensures that means, variances, and correlations (with other variables) can be reliably compared across age, because the indicators are measuring the same thing at different ages. Thus far, a few studies using lab‐based tasks to measure inhibitory control in the preschool years have reported evidence for longitudinal measurement invariance (Hughes, Ensor, Wilson, & Graham, 2009; Wiebe, Espy, & Charak, 2008), or partial (i.e., incomplete) measurement invariance (Willoughby, Wirth, & Blair, 2012). Whether parent reports of inhibitory control show longitudinal measurement invariance is still unclear. In the only study we know of that tested measurement invariance of the CBQ across age, Frohn (2017) reported that 8 out of 13 items of the inhibitory control scale were either deemed not applicable (NA) by a large proportion of parents, or not invariant when comparing a group of 3‐ to 4‐year‐old children with a group of 6‐ to 7‐year‐old children. These items involved: (a) ability for games like "Simon Says," "Mother, May I?", and "Red Light, Green Light,” (b) lowering voice upon request, (c) resisting temptation upon request, (d) preparation for trips and outing by planning, (e) difficulty with waiting in line, (f) difficulty with sitting still upon request, (g) ability for resisting to laugh or smile when this is inappropriate, and (h) difficulty in being careful and cautious when crossing a street. These cross‐sectional results should be replicated in studies with a longitudinal design, to examine whether parent‐reported inhibitory control conceptually changes across development within the same children.

## Parenting and the Development of Inhibitory Control

Various theories emphasize the importance of parenting in the development of higher order skills, including inhibitory control (Rothbart, Sheese, Rueda, & Posner, 2011; Sroufe, 2000). An important theory in this regard is attachment theory. Attachment theory underscores the importance of sensitive and nonintrusive parenting. Sensitive parenting entails that parents notice the cues of their child, interpret these cues correctly, and respond promptly and appropriately (Ainsworth, 1969). Such prompt and appropriate responses support children in staying well‐regulated in the moment, but they should also provide an example of appropriate regulatory strategies which can be internalized (Sroufe, 2000). Intrusiveness entails parental behaviors that are overdirecting, overstimulating, or that interfere with children’s own activities (Biringen, Robinson, & Emde, 2008). These intrusive behaviors relate to increased stress in young children. For instance, higher levels of intrusive parenting are associated with increased levels of cortisol and alpha amylase (Taylor et al., 2013).

The attachment relationship with caregivers is considered to be the model for learning self‐regulation at a physiological level (Perry, Blair, & Sullivan, 2017) and at a behavioral level (e.g., Sroufe, 2000). Although the foundation for this model is laid in infancy, the attachment relationship continues to play an important role in self‐regulation throughout childhood (Sroufe, 2000) and adolescence (Zimmermann, Mohr, & Spangler, 2009). Considering the preschool years, when self‐regulation is still developing, caregivers must give their child the opportunity to master difficult circumstances but also provide support when needed (Sroufe, 2000). The preschool years form a sensitive period for maternal support to affect developmental trajectories of hippocampal volume, a region that has an important function in physiological stress responses (Luby, Belden, Harms, Tillman, & Barch, 2016). Additionally, children from parents who are sensitive and nonintrusive may benefit more from socialization efforts that may further enhance their inhibitory control. A secure attachment with caregivers is found to amplify positive effects of children’s receptive stance toward parental rules, and has been marked as a catalyst for future positive socialization processes (Kochanska et al., 2010). On top of that, children with poor inhibitory control may also tax parent’s ability to remain sensitive and refrain from intrusive behavior, for instance because they show higher levels of noncompliance (Gauvain & Perez, 2008; Morasch & Bell, 2011). This can result in back‐and‐forth processes between parents and children that accumulate over time, also known as developmental cascades (Masten & Cicchetti, 2010). Therefore, children of sensitive and nonintrusive parents are expected to demonstrate more growth in inhibitory control.

A meta‐analysis published 14 years ago reported that there was no concurrent association between parental responsiveness, which included measures of parental sensitivity and inhibitory control (Karreman, Van Tuijl, van Aken, & Deković, 2006). This conclusion was based on a few studies (*n*_studies_ = 7) that examined concurrent associations between responsiveness and a slightly broader inhibitory control construct that also included anxiety‐related behavioral inhibition. Karreman et al. (2006) also reported that negative control, including intrusive behavior, was not associated with inhibitory control (*n*_studies_ = 7). Although the authors tentatively concluded that parental responsiveness and negative control may not be that important for the development of inhibitory control, a growing body of research since then indicates that this conclusion cannot yet be made. For instance, Bernier, Carlson, and Whipple (2010) reported that maternal sensitivity and autonomy support, which can be seen as the opposite of intrusive behavior, at 12–15 months were related to lab performance on inhibitory control tasks at 26 months—although these parenting measures were not found to predict longitudinal change in inhibitory control. In addition, restricting infants behavior at 8 months, for instance by taking objects away and prohibiting, was found to predict lower levels of (lab‐based task) inhibitory control at 8 years (Olson, Bates, Sandy, & Schilling, 2002). Most studies on parenting and inhibitory control are based on only one or two assessments of inhibitory control. An exception is a longitudinal three‐wave study, demonstrating that positive behavior support (e.g., providing structure and positive reinforcement) was linked to faster growth in parent‐reported inhibitory control from 2 to 4 years of age, but not to initial levels of inhibitory control. Moreover, harsh intrusive parenting was linked to lower initial levels of parent‐reported inhibitory control, but not to change in inhibitory control (Moilanen et al., 2010).

In addition, studies on broader self‐regulation constructs, which generally include inhibitory control, demonstrated that higher parental sensitivity predicts higher levels of self‐regulation in toddlers and preschoolers, even when controlling for prior levels of self‐regulation (Blair, Raver, & Berry, 2014; Spinrad et al., 2007). A longitudinal study following children from 2.5 to 4.5 years of age showed that intrusive parenting longitudinally predicted effortful control, again even when controlling for prior levels of effortful control (Eisenberg, Taylor, Widaman, & Spinrad, 2015). On the other hand, Eisenberg et al. (2010) reported that supportive parenting was important for effortful control between 18 and 30 months, but not for 42‐month‐old children’s effortful control. Overall, the available evidence indicates that sensitive and nonintrusive parenting may bolster the development of inhibitory control, but there are only a few studies to date that have related parenting to developmental changes in inhibitory control over more than two measurement occasions.

## Fathers and Mothers

Traditionally, most research into the association of parenting behavior and inhibitory control has focused on mothers. However, a variety of theorists claim that fathers and mothers may play different roles in raising their children, and argue that, whereas mothers typically provide support to their children by comforting them (i.e., the traditional attachment relationship), fathers offer security in situations that are challenging and stimulating (Grossmann, Grossmann, Kindler, & Zimmermann, 2008; Paquette, 2004). These differences in roles imply different ways in which mothers and fathers promote the development of their children’s inhibitory control. Mothers may typically stimulate the development of autonomous regulation by providing support during moment of child distress, whereas the interactions with fathers generally come with a broader range of arousal intensities to practice regulation (Parke et al., 2004). More broadly, as mothers on average still spend two to three times more time on child care than fathers in most Western countries (Huerta et al., 2013), maternal parenting may also have more impact on children’s development compared to fathering.

Supporting these lines of reasoning are studies reporting that the parenting behaviors of mothers and fathers that are associated with children’s self‐regulation (parent reports and lab tasks) differ on average (Karreman, Van Tuijl, Van Aken, & Deković, 2008; Tiberio et al., 2016) and studies reporting that parenting practices of mothers are more strongly or consistently associated with children’s self‐regulation, measured with questionnaires or lab tasks (e.g., Kim & Kochanska, 2012; Towe‐Goodman et al., 2014). Notably, in one of the few studies to consider both mothers and fathers, higher maternal positive control, including sensitivity, and lower paternal negative control were found to promote self‐regulation in preschoolers (Karreman et al., 2008). Hence, whereas maternal sensitivity may be particularly important for the development of children’s self‐regulation, the most important task for fathers when promoting their children’s self‐regulation may be to avoid intrusive behaviors. However, it should also be noted that roles of comforting and activating are not necessarily bound to be fulfilled by mothers and fathers, respectively (e.g., Roggman, 2004). In fact, other studies indicate that parenting practices of mothers and fathers are quite similarly related to their preschooler’s self‐regulation (Bridgett et al., 2018; Kochanska, Aksan, Prisco, & Adams, 2008).

## Current Study

The first objective of the current study was to model the development of inhibitory control between the ages of 2.5 and 6.5. As a prerequisite, we first examined longitudinal measurement invariance, to test the conceptual similarity of the inhibitory control concept across age. With regard to the development of inhibitory control, we expected to find a decelerating increase in inhibitory control over the preschool years. We also explored sex differences in the development of inhibitory control, as girls are generally found to have higher scores on the CBQ inhibitory control scale (Else‐Quest, Hyde, Goldsmith, & Van Hulle, 2006), but growth rates may be similar across sexes (Moilanen et al., 2010). The second objective was to examine whether maternal and paternal sensitivity and intrusiveness at 2.5–3.5 years predicted the initial level and growth of inhibitory control. We expected higher parental sensitivity and lower parental intrusiveness to be related higher initial levels and faster growth of inhibitory control. Lastly, we explored whether mothers’ and fathers’ sensitivity and intrusiveness were similarly, or differently, related to initial levels and growth in inhibitory control, and whether this differed for boys and girls.

## Method

### Sample

This study makes use of data from the longitudinal study Boys will be Boys, focused on gender‐differentiated socialization in the first years of life (see Endendijk et al., 2013). Between April 2010 and May 2011, families were selected from municipality records, and invited by mail to participate in the study. For this study, families with two children in the Netherlands, of which the firstborn child was between 2.5 and 3.5 years old, and their sibling was around 12 months of age old were eligible. Only data considering the firstborn child was used, as inhibitory control in the younger children was measured with differing age‐appropriate questionnaires across waves.

Single parents, families in which either a child or parent had a severe physical or intellectual handicap, and parents born outside the Netherlands or not speaking the Dutch language were excluded from participation. In total, 390 (31%) of the 1,249 contacted families agreed to participate. These families did not differ from the non‐participating families on age, educational level of both parents, and degree of urbanization of the place of residence (all *p*s > .10). A total of 383 families provided data on their children’s inhibitory control during at least one wave and therefore were included in the current study. A total of 373 parents reported on their child’s inhibitory control at Wave 1, whereas 329 (88.20%) parents did so during Wave 4. Little’s missing completely at random test indicated that data were missing completely at random, χ^2^(104) = 107.57, *p* = .385. Follow‐up analyses indicated that the number of missings on inhibitory control was unrelated to parental sensitivity and intrusiveness, age and sex of the child, and maternal and paternal education.

Children (52.5% boys) were on average 3.01 years old (*n* = 383, *SD* = 0.30, range = 2.46–3.61) during the first wave, 4.01 years (*n* = 384, *SD* = 0.30, range = 3.43–4.64) during the second wave, 5.04 years (*n* = 372, *SD* = 0.30, range = 4.44–5.85) during the third wave, and 6.03 years (*n* = 370, *SD* = 0.30, range = 5.50–6.66) during the fourth wave. Mothers were aged between 23.64 and 45.62 years (*M* = 33.95, *SD* = 3.93) and fathers were between 25.84 and 62.97 years of age (*M* = 36.79, *SD* = 5.03). Most participating parents were married, had a cohabitation agreement or registered partnership (93.00%), and the remaining 7.00% lived together without any kind of registered agreement. With regard to educational level, most of the mothers (79.40%) and fathers (76.80%) had a high educational level (academic or higher vocational schooling). This is higher than the national average (i.e., 41.2% of the Dutch population between the age of 25 and 55 was higher educated in 2018; CBS‐statline, 2019).

### Procedure

During four annual measurement waves, each family was visited twice at home, separated by a period of approximately 2 weeks: once with the father, the target child and the younger sibling, and once with the mother and both children. The order in which parents were visited and the order in which they interacted with the firstborn child and the younger sibling was counterbalanced. Both parents were requested to individually complete questionnaires before the first home visit of each wave. During the home visits, parent–child interactions and sibling interactions were video recorded, and the children and both parents completed computerized tasks. The home visits were conducted by pairs of trained undergraduate and graduate students. Informed consent was obtained from all participating families. Families received a payment of 30 Euros and small presents for the children. Ethical approval for this study was provided by the Commission Research Ethics Code of the Leiden Institute of Education and Child Studies.

### Measures

#### Inhibitory Control

The Inhibitory Control subscale of the CBQ (Rothbart et al., 2001) was administered during all four waves. The original subscale contained 13 items, which parents had to answer on a scale from 1 (*never*) to 7 (*always*). This is in contrast to the original rating scale, which ranges from 1 (*extremely untrue of child*) to 7 (*extremely true of child*). Parents could also indicate that an item was NA. The scale used in the current study aligns with some of the other questionnaires based on Rothbart’s work, such as the Early Childhood Behavior Questionnaire (Putnam, Gartstein, & Rothbart, 2006). This rating is also preferred because it is focused on the quantity of behavior, which may be easier to answer for parents, and more suitable to track mean‐level differences than the original rating scale. Adjusting the rating scale of the CBQ has been proposed before (Frohn, 2017). One item was removed because more than 20% of parents indicated that this item was NA across all waves: “My child is able to resist laughing or smiling when it isn't appropriate.” Although a part of the sample (*n* = 200) was younger than the intended age range of the CQB, that is, 3 years of age, this could not explain this high percentage: a high percentage of parents of children older than 3 years also indicated that this item was NA (see Table S1). Across all four waves, mean scores of father and mother reported inhibitory control were sufficiently correlated (*r *= .46–.53). Items were generally also sufficiently correlated (*r *= .15–.40). To create more robust scores for inhibitory control, father and mother reports were averaged at the item level for subsequent factor analyses as described in the results section. Cronbach’s alpha using these averaged items indicated that the internal consistency of the scale was good, ranging from .77 to .85 across waves.

#### Parenting

Parents and children were videotaped after they were asked to play with a bag with toys for 8 min. Parental sensitivity and nonintrusiveness were coded by a team of seven coders using the fourth edition of the Emotional Availability Scales (EAS; Biringen et al., 2008). Sensitivity refers to the parent’s ability to be warm and appropriately responsive to the child, whereas nonintrusiveness indicates the parent’s ability to give the child space to explore and to refrain from intrusions on the child’s activities. Both dimensions were divided into seven subscales, in which the first two subscales were coded on a 7‐point Likert scale and the other subscales are coded using a 3‐point Likert scale (potential score range 7–29). Fathers and mothers received a global rating score for both sensitivity and nonintrusiveness based on their behavior during the entire 8‐min free play session. For the Nonintrusiveness scale, one subscale (the adult is made to “feel” or “seem” intrusive) was excluded because it refers to child behavior rather than parental behavior, which resulted in a potential score range of 7–26 (Hallers‐Haalboom et al., 2014). Interrater reliability, determined on 15% of the participating families, was sufficient, with a mean intraclass correlation coefficient for sensitivity of .81 (range = .73–.92) and .84 (range = .76–.93) for nonintrusiveness. In addition, the first 100 videotapes were coded twice by separate coders, and regular meetings were organized to prevent coder drift. Nonintrusiveness was reverse coded so that higher scores represented more intrusiveness.

### Plan of Analyses

All analyses were conducted using M*plus* 8.0 (Muthén & Muthén, 2012), using robust maximum likelihood (MLR) estimation, and full information maximum likelihood to account for missing data.

#### Measurement Invariance

We first fitted a one‐factor model on the data of the first wave, to test whether all items of the inhibitory control scale loaded on a single factor for parent reports (mother and father reports collapsed for each item). Next, we examined measurement invariance. We first constrained factor loadings over time (i.e., metric invariance), followed by the intercepts (i.e., scalar invariance) and the residuals (i.e., error variance invariance). Potential sources of invariance were detected by inspecting the Modification Indices (MI) in M*plus*. Partial scalar invariance is required to compare means over time (Little, 2013).

Model fit was evaluated through the comparative fit index (CFI), Tucker–Lewis index (TLI), and root mean square error of approximation (RMSEA). CFI and TLI values above .90 and RMSEA values below .08 indicate a sufficient fit. In addition, ΔCFI between the restricted and less restricted model had to be < .01 (Cheung & Rensvold, 2002). The corrected chi‐square and corrected chi‐square difference test implemented in M*plus* were also reported. However, as these tests are known to be too sensitive to small and unimportant deviations, we do not rely heavily on these indices. When the absolute model fit and ΔCFI were sufficient, we were very conservative with applying any further changes to the model. Any further changes of the model either had to be supported by a large MI, and/or had to be logical from a theoretical point of view.

Subsequently, we reran the factor analysis using the effect coding method as proposed by Little, Slegers, and Card (2006). In this method, the set of loadings are constrained to average to 1, and the set of indicator intercepts are constrained to sum up to 0. This method results in estimated latent means and variances that reflect the observed metric of the underlying items. As such, this method provides meaningful latent means and variances, and is therefore particularly useful for analyses in which the means of latent constructs are of interest. The resulting scores from this analysis were saved for subsequent analyses.

#### Development of Inhibitory Control and Associations With Parenting

Because we were interested in sex differences in growth of inhibitory control, we fitted univariate growth models on the saved factor scores of inhibitory control for boys and girls separately, to determine whether the shape of growth was similar for boys and girls (Duncan, Duncan, & Strycker, 2006). We compared a latent intercept model, a linear growth model, and a quadratic growth model. If the models for boys and girls resulted in a similar growth shape, we conducted a multi‐group growth model with sex as grouping variable. We compared a model in which parameters were restricted across sex with a freely estimated model. If the restricted model fitted the data better, we also consulted MIs to determine if single parameters could be released. If the shape of growth differed between boys and girls, we conducted separate growth models for boys and girls.

Because children varied substantially in age within the measurement waves, we estimated growth models with individual varying times of observation (i.e., the TSCORES option in M*plus*). This approach avoids biases in growth factor variances that could potentially occur when fixed time intervals are applied to age heterogeneous samples (Mehta & West, 2000). Lastly, we included maternal and paternal sensitivity and intrusiveness as predictors in the final growth model(s). Both maternal and paternal parenting variables were added in the model simultaneously. We examined whether maternal and paternal sensitivity and intrusiveness were similarly related to the development of inhibitory control for both boys and girls. Specifically, we tested four models: children’s sex unconstrained and parents’ sex unconstrained, children’s sex unconstrained and parents’ sex constrained, children’s sex constrained and parents’ sex unconstrained, and children’s sex constrained and parents constrained. Due to the multilevel structure that defines the TSCORES option in M*plus*, standardized coefficients were not available. For all models, we therefore reported the unstandardized coefficients.

Common fit indices are not provided when using the TSCORES option. Therefore, only the Akaike Information Criterion (AIC) and Bayesian Information Criterion (BIC) were used to compare model fit for the growth models. Higher BIC and AIC values indicate a worse model fit for the restricted model. As the BIC more strongly penalizes model complexity, this fit index was considered superior to the AIC. Decreases in BIC values larger than 10 indicate serious model improvements (Raftery, 1995).

## Results

### Measurement Invariance

At Wave 1, a one‐factor model showed near sufficient fit to the data (χ^2^(35)* *= 150.668, *p *< .001, RMSEA* *= .069, CFI* *= .906, TLI* *= .885). Based on the largest MI, we allowed for covariance between the residuals of two highly similar items ("Has a hard time following instructions" and "Is good at following instructions"). This model fitted the data sufficiently (χ^2^(53)* *=  119.114, *p *< .001, RMSEA* *= .058, CFI* *= .936, TLI* *= .920). Standardized factor loadings ranged from .10 to .74.

Table 1 shows the fit indices of the tested models for measurement invariance. The configural model, in which both factor loadings and intercepts were freely estimated across waves, showed a sufficient fit to the data. The metric model also showed a sufficient fit to the data, as evident by CFI, TLI and RMSEA statistics as well as a ΔCFI of < .01. The scalar model showed a poor fit to the data, as evident by CFI and TLI below .90. In addition, the ΔCFI of .04 also indicated that imposing scalar invariance (i.e., similar intercepts across waves) resulted in a worse model fit.

**Table 1:** Fit Indices for the Models Testing Measurement Invariance

	χ^2^	*df*	*p*	CFI	TLI	RMSEA	Δχ^2^
1. Configural model	1,529.016	998	< .001	.922	.912	.037	
2. Metric invariance	1,577.929	1,031	< .001	.919	.912	.037	2 versus 1 (33): 48.92 *p *= .037
3. Scalar invariance	1,907.290	1,064	< .001	.876	.868	.045	3 versus 2 (33): 338.56 *p* < .001
4. Adjusted scalar invariance	1,664.400	1,058	< .001	.911	.905	.039	4 versus 2 (27): 88.88, *p* < .001
5. Residual invariance	1,730.072	1,088	< .001	.905	.902	.039	5 versus 4 (30): 61.39, *p *= .001

Note

CFI = comparative fit index; TLI = Tucker–Lewis index; RMSEA = root mean square error of approximation.

Modification Indices indicated that the model could be substantially improved by releasing various intercepts. We released parameters with the highest MI one by one until the model had a sufficient fit. A model in which six intercepts of five items were released showed a sufficient fit to the data, see Table 1. In addition, ΔCFI was < .01.

Lastly, we tested whether the items that were scalar invariant were also invariant with respect to their residuals. As can be seen in Table 1, imposing residual invariance resulted in a sufficient model fit and a ΔCFI of < .01. Thus, the inhibitory control scale was found to be partially invariant over time. Table 2 lists an overview of the items, with their factor loadings, intercepts, and psychometric concerns. We reran the factor analyses using the effect coding method as proposed by Little et al. (2006), and saved the factor scores for subsequent analyses. The resulting descriptive information and correlations are shown in Table 3.

**Table 2:** Overview of Items and Psychometric Concerns

Item	W1	W2	W3	W4	Factor loading	Intercept	Intercept Wave 1/Wave 2
Can lower voice					.56	4.84	
Good at games like "Simon Says"	1, 2	1^a^, ^*^, 2			.19	5.63	4.73/5.22^b^, ^**^
Hard time following instructions (*R*)					.62	5.07	
Prepares for trips and outings by planning	1, 2	1			.25	3.98	3.41/—
Can wait before entering into new activities					.65	4.64	
Difficulty waiting in line (*R*)					.59	4.43	
Trouble sitting still (*R*)	2				.37	5.40	4.93/—
Able to resist laughing while inappropriate^c^	1	1	1	1	—	—	—
Good at following instructions					.61	5.08	
Approaches dangerous places slowly and cautiously					.33	5.70	
Not careful and cautious in crossing streets (*R*)	2				.28	5.26	4.80/—
Can easily stop an activity					.74	4.81	
Able to resist temptation	2				.63	4.77	4.51/—

Note

1 = more than 20% not applicable (NA) response, 2 = not scalar invariant. W = wave.

^a^ Only mother reports had more than 20% NA responses.

^b^ Wave 2.

^c^ Item was removed.

**Table 3:** Correlations and Descriptive Information

	*N*	*M* (*SD*)	Range	1	2	3	4	5	6	7
1. Maternal sensitivity	389	24.92 (2.77)	11.00–29.00							
2. Maternal intrusiveness	389	20.36 (3.40)	9.00–26.00	−.56^b^, **						
3. Paternal sensitivity	390	24.04 (2.30)	11.00–29.00	.19^b^, **	−.14^b^, **					
4. Paternal intrusiveness	390	19.66 (3.46)	9.00–26.00	−.11^a^, *	.13^a^, *	.52^b^, **				
5. Inhibitory control Wave 1	373	4.74 (0.46)	3.01–5.75	.16^b^, **	−.00	.11^a^, *	−.10^a^, *			
6. Inhibitory control Wave 2	350	5.01 (0.52)	2.63–6.15	.07	.02	.07	−.10	.85^b^, **		
7. Inhibitory control Wave 3	334	5.10 (0.55)	2.61–6.40	.08	−.03	.07	−.12^a^, *	.78^b^, **	.82^b^, **	
8. Inhibitory control Wave 4	329	5.09 (0.55)	3.06–6.44	.05	−.02	.14^a^, *	−.15^b^, **	.76^b^, **	.81^b^, **	.87^b^, **

Note

Factor scores were calculated using the effect coding method as proposed by Little et al. (2006).

* *p* < .05.

** *p* < .01.

Corrections added on September 25, 2020, after first online publication: the indicator alphabets "a" and "b" are removed from the table.

### Development of Inhibitory Control

Separate growth models for boys and girls indicated that both boys and girls exhibited change in inhibitory control (see fit indices in Table 4), as a linear model fitted the data better than a model with only an intercept. In addition, for both boys and girls, BIC and AIC values indicated that a quadratic growth model fitted the data better than a linear model. In these models, there was no significant variance around the linear slope and quadratic slope. We restricted the variance of the quadratic factor to zero, which is a common procedure as the variance of the quadratic slope can rarely be estimated (Tofighi & Enders, 2008). The adjusted quadratic models showed a relatively similar fit to the data compared to the initial quadratic models. In this model, there was significant variance around the intercept and the linear slope. In all subsequent analyses, the variance around the quadratic slope was set to zero.

**Table 4:** Fit Indices for the Growth Models

	Girls		Boys		Total	
	BIC	AIC	BIC	AIC	BIC	AIC
Intercept	538.629	519.405	738.596	718.776	1,268.646	1,244.958
Linear model	348.690	319.854	617.813	588.083	960.612	925.080
Quadratic model	323.955	282.303	566.711	523.768	868.444	817.119
Adjusted quadratic model	313.026	280.986	554.069	521.036	856.311	816.831

After determining the shape of growth, multi‐group analyses showed that a model in which the intercepts, means, variances, and covariances of the growth factors were constrained across children’s sex (BIC: 856.311, AIC: 816.831) showed lower BIC values and higher AIC values than a model in which all parameters were released (BIC: 880.983, AIC: 802.022). In addition, there were no MIs that resulted in a better fit of the model. We therefore concluded that the development of inhibitory control was not only similar in shape but also in initial level, rate, and direction of development for boys and girls. Inhibitory control showed an average increase that decelerated over time, with variance around the intercept and linear slope (intercept* *= 4.59, *p *< .001, σ* *= .20, *p *< .001, linear slope* *= 0.33, *p *< .001, σ* *= .01, *p* < .001, quadratic slope* *= −0.05, *p *< .001). The intercept and slope were not significantly correlated, −.01, *p *= .235, indicating that the initial level was not associated with the rate of change in inhibitory control.

### Associations With Parenting

We next examined associations between the intercept and linear slope of inhibitory control and parenting (associations between parenting and the quadratic slope were not estimated as we fixed the variance of this slope to zero). A model in which maternal and paternal sensitivity and intrusiveness were similarly associated with boys’ and girls’ initial level and slope of inhibitory control showed the best fit to the data (see Table 5). There were no MIs indicating that releasing any of the parameters would result in a better fit. Thus, parenting practices of mothers and fathers are similarly related to the initial level and growth of inhibitory control for both boys and girls.

**Table 5:** Fit Indices for the Growth Models with Parenting

	Total	
	BIC	AIC
Children unconstrained parents unconstrained	912.534	809.953
Children unconstrained parents constrained	881.885	810.868
Children constrained parents unconstrained	876.387	805.370
Children constrained parents constrained	865.436	810.200

**Figure 1:**
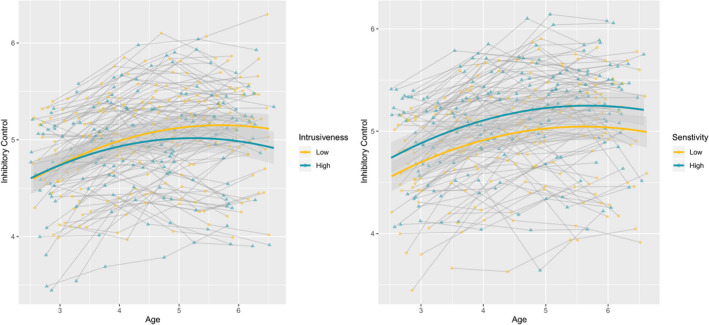
Growth curves and individual trajectories for inhibitory control at at least 1 sd below and above the mean of parental intrusiveness (left) and sensitivity (right). Shaded areas represent 95% confidence intervals. [Color figure can be viewed at wileyonlinelibrary.com]

Higher parental sensitivity was related to a higher initial level of inhibitory control (0.020, *SE *= .006, *p *= .001). However, parental sensitivity was unrelated to growth in inhibitory control (−0.002, *SE *= .002, *p *= .161). In addition, higher intrusiveness did not predict the initial level of inhibitory control (0.007, *SE *= .005, *p* = .194), but did predict slower growth of inhibitory control (−0.004, *SE *= .001, *p *= .005). Figure 1 illustrates that children with relatively sensitive parents consistently showed higher levels of inhibitory control compared to children with relatively insensitive parents, but the rate of change did not differ. On the other hand, children of relatively intrusive parents did not differ in the initial level of inhibitory control, but they did show a slower rate of development compared to children of less intrusive parents.

## Discussion

In the current longitudinal multi‐method study, we examined the development of parent‐reported inhibitory control between 2.5 and 6.5 years of age, and tested associations with both maternal and paternal sensitivity and intrusiveness. We found that the inhibitory control scale demonstrated partial longitudinal measurement invariance. As expected, inhibitory control showed an increase that decelerated over time. This development was similar for boys and girls. Whereas higher parental sensitivity was related to a higher initial level of inhibitory control, higher parental intrusiveness was related to a slower rate of development. These associations were similar for mothers and fathers.

### Longitudinal Measurement Invariance and Development

Knowledge regarding longitudinal measurement invariance of inhibitory control, that is, whether a scale functions similarly across age, is a critical first step before mean‐level development can be modeled. When a scale is not invariant across time, observed longitudinal changes are likely confounded by properties that are not the construct of interest. Despite the popularity of the CBQ, longitudinal measurement invariance has rarely been tested for this questionnaire. We tested whether the CBQ inhibitory control scale was invariant between the ages of 2.5 and 6.5 years. The results of the current study show that all items of the inhibitory control scale were metric invariant. This indicates that the quality of the items as a reflection of inhibitory control does not change over time (Little, 2013). Items that were a good indicator of inhibitory control (i.e., with a high factor loading) were generally those that addressed children’s compliance to requests. For instance, “can stop an activity when s/he is told ‘no’” had the highest factor loading. Items that were a poor indicator of inhibitory control were those that addressed relatively mature and independent behavior, such as crossing streets carefully. It should be noted that one item (i.e., able to resist laughing while inappropriate) was removed prior to invariance testing, because a high percentage of parents noted that this question was NA to their child. Although our sample contained children who were up to half a year younger than the intended age range of the scale (i.e., younger than 3–7 years), this could not explain the high percentage of NA responses. Situations in which children are not supposed to laugh apparently happen too infrequently to assess inhibitory control.

Five items of the inhibitory control scale did not demonstrate scalar invariance across time. Longitudinal scalar invariance indicates that children with the same level of inhibitory control have the same scores on the underlying item, irrespective of their age (Little, 2013). For most items of the inhibitory control scale, this appeared to be the case. However, to have the same level of inhibitory control as children in Waves 2, 3, and 4, children in Wave 1 had to be less well capable of preparing for trips, sitting still, carefully crossing streets, and resisting temptation. In addition, in Waves 1 and 2, children had to be less good at games such as Simon says to receive the same score on inhibitory control, compared to Waves 3 and 4. With one exception (able to resist temptations), these items also contributed quite poorly to inhibitory control, that is, they also had a low factor loading, indicating that these items are not a strong and stable indicator of inhibitory control. The items that demonstrated scalar invariance were also invariant with respect to their residuals. Although this is not a requirement for comparing means (Little, 2013), it does demonstrate that the items used to assess inhibitory control are equally reliable across age.

The results of this study are in line with a previous study comparing younger and older children on various CBQ scales, reporting that 8 of the 13 inhibitory control items were either deemed NA by a large proportion of parents, or showed either metric or scalar invariance (Frohn, 2017). The same items were flagged as problematic in the current study, except for two (assessing difficulty waiting in line and lower voice when asked to do so) which were invariant in the current study, but not in the study by Frohn (2017). The overlap in results shows that there may be a consistent set of problematic items in the inhibitory control scale. Yet, although various guidelines have been proposed to determine adequate measurement invariance, Little (2013) proposed that at least partial scalar invariance is required to examine mean‐level changes. Therefore, there was sufficient ground to examine mean‐level development of inhibitory control.

We found a modest increase in inhibitory control between the ages of 2.5 and 6.5 years. In agreement with previous research, this increase decelerated over time (Chang et al., 2014). We found no evidence for differences between boys and girls in initial level of inhibitory control. This is contrast to previous research findings that girls score higher than boys on inhibitory control (Else‐Quest et al., 2006; Moilanen et al., 2010). This divergent result may be explained by the relatively high educational level that characterizes most participants in the current study. Educational level has been associated with less traditional views on gender roles of parents (e.g., Jan & Janssens, 1998), which can subsequently result in smaller gender differences. In alliance with previous research, children’s sex was not related to growth rates in inhibitory control (Moilanen et al., 2010). A remaining question concerns how development of inhibitory control on lab tasks and parent reports are related to each other. Future studies could therefore examine the development of inhibitory control using both lab tasks and parent reports.

### Parenting and the Development of Inhibitory Control

In keeping with theoretical work (Ainsworth, 1969; Rothbart et al., 2011; Sroufe, 2000), parental sensitivity was associated with a higher initial level of inhibitory control. Interestingly, parental sensitivity was not related to growth in inhibitory control. Hence, this study demonstrates that prompt and appropriate responses predict the level of inhibitory control at age 2.5 (i.e., the age of the youngest child during the first assessment), but does not support the premise that sensitivity enhances the development of inhibitory control after the age of the first assessment. These results are in contrast to previous work (Moilanen et al., 2010), reporting that positive behavior support, including sensitivity, did promote the development of inhibitory control. A possible explanation for these discrepant findings is that Moilanen et al. (2010) examined positive behavior support, which also involved providing structure. This aspect of parenting may be more important in the development of self‐regulation across the preschool years than sensitivity (Karreman et al., 2006).

Higher levels of parental intrusiveness were associated with a slower increase in inhibitory control. Parents who show high levels of intrusive behaviors may deprive their children of opportunities to practice and improve autonomous regulation skills. The findings of this study are in line with research demonstrating that high levels of parental directiveness when children are 3.5 years old negatively predict cognitive functioning at 4.5 years of age (Landry, Smith, Swank, & Miller‐Loncar, 2000), and with research reporting that restrictive parenting at 8 months predicted lower levels of (lab based task) inhibitory control at 8 years (Olson et al., 2002). The study adds to literature by demonstrating that intrusiveness predicts a slower increase in inhibitory control. Overall, intrusive parenting may leave children ill‐equipped for showing independent self‐regulation later in development.

In line with previous work (Karreman et al., 2006; Moilanen et al., 2010), the associations between parenting and (growth in) inhibitory control were modest. Without question, the development of inhibitory control is affected by other processes on various levels, such as brain maturation and language development (e.g., Wolfe & Bell, 2007). Yet, whereas the current study demonstrated how parenting predicts children’s development, other studies have demonstrated that the behaviors that come with poor child inhibitory control, such as noncompliance, also tax parent’s ability to remain sensitive and nonintrusive (e.g., Gauvain & Perez, 2008). This can result in back‐and‐forth processes that accumulate over time (Masten & Cicchetti, 2010). Such cascading processes may be prevented by intervening on parenting behavior early on.

### Differences Between Mothers and Fathers

The associations between sensitivity and intrusiveness on the one hand, and both the initial level and development in inhibitory control on the other hand, were similar in strength for mothers and fathers. This was also irrespective of children’s sex. The results indicate that fathers and mothers equally contribute to the development of their children’s inhibitory control, which is in line with a variety of previous studies focused on concurrent associations between parenting and inhibitory control (Bridgett et al., 2018; Towe‐Goodman et al., 2014), but in contrast to other studies (Karreman et al., 2008; Kochanska et al., 2008; Tiberio et al., 2016). Thus far, only a few studies have considered the contribution of both mothers and fathers on children’s self‐regulation. It is possible that unique influences of fathers are not easily detected with measures that have been developed from primarily mother‐focused research, like the EAS that was used in the current study. For instance, although a qualitative study demonstrated that mothers and fathers differed on a variety of parenting behaviors (e.g., mothers tended to be more directive, and engaged in empathic conversations, whereas fathers followed children’s lead, engaged in physical play, and challenged children), fathers and mothers did not differ on the EAS sensitivity and nonintrusiveness scales (John, Halliburton, & Humphrey, 2013). Future studies on the role of mothers and fathers in the development of inhibitory control may benefit from including a broader array of parenting behaviors, such as challenging parenting behavior that playfully encourages children to push their limits (Majdandžić, Möller, de Vente, Bögels, & van den Boom, 2014).

### Limitations and Future Directions

Despite various strengths, such as the use of multiple informants to assess inhibitory control, the rigorous testing of measurement invariance, and the usage of observed parenting measures, this study also has some limitations. First, participating families generally had a high socio‐economic status, and consisted of a traditional family constellation (i.e., two parents and two children). The current study therefore adds to the literature on the development of inhibitory control, which has primarily focused on children at risk. However, future studies should examine the development of inhibitory control, and the role of parents in this development, in a more representative sample and in less traditional family compositions (single‐parents, same‐gender parents). Second, we could not examine associations between growth in parenting behaviors and development in inhibitory control, as parenting was not assessed during all four waves. Future studies should examine how the development of parenting relates to their children’s inhibitory control development. Third, although a major strength of the current study is the inclusion of both mothers and fathers, we did not observe mothers and fathers simultaneously. Previous work has shown that co‐parenting is related to children’s effortful control over and above maternal and paternal parenting (Karreman et al., 2008). A next step for future research to take is to examine how co‐parenting relates to the development of inhibitory control. Fourth, the design of this study did not permit us to examine genetic and biological factors that most likely play a role in the association between parenting and (growth of) inhibitory control. It is very well possible that the link between parenting and inhibitory control is at least partially explained by shared genes and/or shared environment. Lastly, we were not able to control for the involvement of mothers and fathers in the caregiving of their children, whereas this factor may be more important than parental sex in predicting the development of inhibitory control.

### Conclusion

Overall, the present study involved a thorough longitudinal examination of the development of inhibitory control. We found parent‐reported inhibitory control to be subjected to conceptual changes in the preschool years, emphasizing the need for researchers to test for longitudinal measurement invariance prior to modeling mean‐level changes. In the current general population study, parent‐reported inhibitory control for both boys and girls showed a decelerating increase in the preschool years. Importantly, parenting behaviors that are related to higher levels of inhibitory control during the first assessment are not predictive for faster increases in inhibitory control. The findings show that parental sensitivity is associated with a consistently higher level of inhibitory control in the preschool years. In addition, to bolster the development of inhibitory control, parents have to give their children space to explore, and interfere sparingly. Mothers’ and fathers’ parenting practices were of similar importance to the development of inhibitory control, suggesting that interventions designed to bolster the development of inhibitory control should target both parents.

## Supporting information


**Table S1.** Number of Parents Answering Non‐Applicable During Four Waves
