# Supplementary material for: Inhibitory Control Across the Preschool Years: Developmental Changes and Associations with Parenting
Source: Child Dev. 2020 Aug 7;92(1):335–50. doi: 10.1111/cdev.13426 (PMC7891350; doi:10.1111/cdev.13426)
Supplement: Supplementary file 1 — Table S1. Number of Parents Answering Non‐Applicable During Four Waves [file CDEV-92-335-s001.docx]

Table S1

*Number of Parents Answering Non-Applicable during Four Waves*

|  | F  W1 | M  W1 | F W2 | M W2 | F W3 | M W3 | F W4 | M W4 |
| --- | --- | --- | --- | --- | --- | --- | --- | --- |
| Able to resist temptation | 1 | 0 | 0 | 0 | 0 | 1 | 0 | 0 |
| Hard time following instructions (R) | 2 | 0 | 0 | 0 | 0 | 0 | 0 | 1 |
| Good at following instructions | 0 | 0 | 1 | 0 | 0 | 0 | 0 | 2 |
| Difficulty waiting in line (R) | 4 | 1 | 0 | 0 | 0 | 0 | 1 | 1 |
| Can easily stop an activity | 0 | 0 | 1 | 1 | 0 | 0 | 0 | 0 |
| Good at games like "Simon Says" | 146 (85) | 181 (111) | 71 | 101 | 26 | 24 | 24 | 17 |
| Prepares for trips and outings by planning | 119 (68) | 121 (78) | 47 | 49 | 17 | 17 | 12 | 7 |
| Can wait before entering into new activities | 5 | 6 | 3 | 3 | 0 | 1 | 0 | 4 |
| Can lower voice | 3 | 5 | 2 | 5 | 1 | 1 | 1 | 2 |
| Not careful and cautious in crossing streets (R) | 26 | 21 | 9 | 4 | 2 | 2 | 2 | 1 |
| **Able to resist laughing inappropriate** | **259 (149)** | **287 (153)** | **165** | **191** | **126** | **135** | **89** | **92** |
| Trouble sitting still (R) | 29 | 24 | 11 | 8 | 7 | 6 | 5 | 2 |
| Approaches dangerous places slowly and cautiously | 9 | 16 | 6 | 4 | 2 | 2 | 5 | 1 |

*Note.* F = Father, M = Mother, W = wave. Values in parentheses represent the number of parents for children under 3 years. Bold item was excluded from subsequent analyses.
